# Polyphenolics and triterpenes presence in chloroform extract of *Dicranopteris linearis* leaves attenuated paracetamol-induced liver intoxication in rat

**DOI:** 10.1186/s12906-020-03200-2

**Published:** 2021-01-14

**Authors:** Zainul Amiruddin Zakaria, Adibah Sahmat, Azfar Hizami Azmi, Amal Syahirah Nur Zainol, Maizatul Hasyima Omar, Tavamani Balan, Lilis Sulistyorini, R. Azizah, Muhammad Nazrul Hakim Abdullah

**Affiliations:** 1 Department of Biomedical Science, Faculty of Medicine and Health Sciences, Universiti Putra Malaysia, 43400 UPM Serdang, Selangor Malaysia; 2 Integrative Pharmacogenomics Institute (iPROMISE), Universiti Teknologi MARA, Puncak Alam Campus, 42300 Bandar Puncak Alam, Selangor, Malaysia; 3 Department of Environmental Health, Faculty of Public Health, Campus C Universitas Airlangga, Jalan Mulyosari, Surabaya Indonesia; 4 Phytochemistry Unit, Herbal Medicine Research Level 5, Block C7, National Institutes of Health (NIH), Setia Alam, 40170, Shah Alam, Selangor, Malaysia; 5 Faculty of Pharmacy and Health Sciences, Universiti Kuala Lumpur Royal College of Medicine Perak, 30450 Ipoh, Perak Malaysia

**Keywords:** *Dicranopteris linearis*, Chloroform extract, Paracetamol intoxication, Hepatoprotective activity, Triterpenes, Hesperetin, Endogenous enzymatic antioxidant system

## Abstract

**Introduction:**

Water-soluble, but not lipid-soluble, extract of *Dicranopteris linearis* leaves has been proven to possess hepatoprotective activity. The present study aimed to validate the hepatoprotective and antioxidant activities, and phytoconstituents of lipid-soluble (chloroform) extract of *D. linearis* leaves.

**Methods:**

The extract of *D. linearis* leaves (CEDL; 50, 250 and 500 mg/kg) was orally administered to rats for 7 consecutive days followed by the oral administration of 3 g/kg PCM to induce liver injury. Blood was collected for liver function analysis while the liver was obtained for histopathological examination and endogenous antioxidant activity determination. The extract was also subjected to antioxidant evaluation and phytochemicals determination via phytochemical screening, HPLC and UPLC-HRMS analyses.

**Results:**

CEDL exerted significant (*p* < 0.05) hepatoprotective activity at 250 and 500 mg/kg and significantly (*p* < 0.05) reversed the PCM-induced decrease in rat’s liver endogenous antioxidant (catalase and superoxide dismutase) level. CEDL possessed a high antioxidant capacity when measured using the ORAC assay, but a low total phenolic content value and radical scavenging activity as confirmed via several radical scavenging assays, which might be attributed particularly to the presence of triterpenes. Phytochemicals screening demonstrated the presence of triterpenes and flavonoids, while UPLC-HRMS analysis showed the presence of polyphenols belonging to the hydroxybenzoic acids, hydroxycinammates and flavonoid groups.

**Discussion and conclusion:**

Lipid-soluble bioactive compounds of CEDL demonstrated hepatoprotective effect against PCM intoxication partly via the modulation of the endogenous antioxidant defense system, and exerted high antioxidant capacity. Further investigation is warranted to identify the potential hepatoprotective leads from CEDL for future drug development.

## Background

Despite its vital role in various physiological processes including maintenance and regulation of homeostasis, and detoxification of drugs and xenobiotics in the body, the liver is widely exposed to injury or toxicity due to direct exposure to various intoxicants such as like chemotherapeutic agents, chronic alcohol consumption and pathogenic microbes [1]. One of the toxicants continuously associated with liver injury is paracetamol (PCM), which is an effective drug for the treatment of pain, inflammatory and fever. At its normal therapeutic doses, PCM can be obtain as an over-the-counter medicine since it is a safe drug for all age groups. However, the intentional or unintentional overdoses of PCM might cause acute damage to the liver, which may lead to fatal liver necrosis if not properly treated. Unfortunately, there are inadequate treatment selections to treat liver necrosis [2].

At therapeutic dose, PCM undergo sulfation and glucuronidation in the liver wherein between 5 and 10% of it is rapidly metabolize into *N*-acetyl-*p*-benzoquinone imine (NAPQI) by the hepatic cytochrome P450 (CYP450) system, mainly CYP2E1. Being a reactive metabolite, NAPQI will form covalent binding with various cellular nucleophiles (e.g. RNA, DNA and proteins), which may result in cell death. Moreover, CYP2E1 also take part in the NADPH oxidase activity leading to the continuous production of free radicals that may result in hepatic injury. Concurrent to this, the glutathione redox system inactivates the formed NAPQI via glutathione conjugation resulting in the diminution of cellular GSH supplies and creation of protein adducts [3, 4].

Excessive stress in the mitochondria resulting from extreme oxidative and nitrosative processes result in the initiation of enzymes of signaling cascade, which disrupts and destabilizes the membrane potential, causing the mitochondria to swell until its membrane rupture. The rupture of mitochondrial membrane will finally lead to the initiation of nuclear DNA fragmentation [5]. Collectively, fragmentation of DNA together with impairment of mitochondrial play major role in hepatocyte necrosis seen in PCM-induced liver injury (PILI). Excessive formation of metabolites reduces GSH level in the liver, thus, PCM intoxication is currently treated using *N*-acetylcysteine (NAC), a glutathione precursor, with aim of replenishing the GSH supplies in the liver. Despite being the only drug permitted to be used a cure for PILI, NAC is only effective following the oral or intravenous administration within 10 h of PCM overdose [6].

To avoid depending on NAC as the only source of antidote for PILI, researchers have increase their effort to search for alternative sources of agents to treat PILI and natural products have been a good source of leads for treating various diseases including liver injury. Natural products, especially plants, have been playing key role in drug discovery in various traditional cultures due to their wide range of diversity of active bioactive molecules [7]. Due to disease-inhibiting capabilities, these compounds are extremely useful as natural drugs that are less toxic, more effective and provide a venue for the development of natural products into the potent drug. For example, polyphenolic compounds have an imperative role in stabilizing lipid oxidation and are associated with antioxidant activity [8].

Since PILI involved oxidative stress and inflammation, it is widely claimed that any compounds that can prevent oxidation and inflammation activities could also exert liver protective action [9]. In this case, medicinal plants have been widely known as the major source of bioactive compounds with antioxidant and anti-inflammatory activities. One of the plants that have been actively studied for its medicinal potentials in our laboratory is *Dicranopteris linearis* L. (Family *Gleicheniaceae*), a type of fern known to the Malay as *‘Resam’*. Other than that, it is important to highlight that *D. linearis* is traditionally used to control fever, to get rid of intestinal worms, to treat boils, ulcers, and wounds [10] whereas, scientifically, the plant has been proved to exert various pharmacological activities including anti-inflammatory [11, 12], hepatoprotective [13] and antioxidant [14] to name a few. The hepatoprotective activity, in particular, was observed using the plant’s methanol extract (MEDL), which has been acknowledged to extract a mixture of polar, non-polar and intermediate polar bioactive compounds. Since MEDL contains mixture of bioactive compounds with different polarities, it is difficult to attribute the hepatoprotective activity to any particular group of bioactive compounds according to their polarity. Taking into consideration that the chloroform extract of *D. linearis* (CEDL) demonstrated anti-inflammatory and antioxidant activities [11, 15], wherein these activities have been reported to play role in the mechanisms of hepatoprotection, the present study was proposed to evaluate the hepatoprotective potential of this hydrophobic/lipid-soluble phytoconstituents (CEDL) of *D. linearis* leaves using the in vivo PILI model in rats.

## Methods

### Plants materials

#### Leaves of ***D. linearis***

The leaves of *D. linearis* were collected within the vicinity of Universiti Putra Malaysia (UPM), Selangor, Malaysia between September to October 2012 based on the previous voucher specimen (SK 1987/11) deposited at the Herbarium of the Institute of Bioscience, UPM, Malaysia.

#### Preparation of chloroform extract of ***D. linearis*** leaves

The leaves of *D. linearis* were air-dried at room temperature and under the shade for approximately 14 days. The dried leaves were then grinded using a sterile electric grinder and the powdered leaves (1 kg) obtained were soaked in chloroform [ratio of 1:20 (w/v)] for 72 h at room temperature. The mixture was then sequentially filtered using cotton wool followed finally by filter paper (Whatman No.1) to obtain the supernatant. The residue collected was then subjected to the extraction and filtration processes for another two times. All supernatant collected were pooled together and then subjected to the evaporation process under reduced pressure at 40 °C to obtain the dried extract [11]. The extract was then prepared in the dose of 50, 250 and 500 mg/kg by diluting it in 8% tween 80 (vehicle). Each dose of CEDL was then assayed for their ability to ameliorate PCM-induced liver damage in rats.

### Analysis of phytoconstituents of CEDL

#### Phytochemical screening of CEDL

The extract, CEDL, was subjected to the phytochemical screening processes to detect the existence of flavonoids, triterpenes, tannins, saponins, alkaloids and steroids. The detail procedure has been described elsewhere [11].

#### Reagents and chemicals for HPLC analysis

The following chemicals were used for the HPLC analysis of CEDL, namely: acetonitrile and formic acid LCMS grade (Fisher Scientific, Kuaa Lumpur, Malaysia), chemical standards (i.e. catechin, caffeic acid ferulic acid) (Sigma Co., NJ, USA) and reverse osmosis Milli-Q water (18.2 MΩ) (Millipore, Billerica, USA). The reverse osmosis water was used for all solutions and dilutions whereas the standards were diluted in methanol/water (v:v, 1:1) to 10 mg/mL and filtered through 0.22 μm membranes before the LC-MS analysis.

#### HPLC analysis of CEDL

The extract, CEDL, was also subjected to HPLC analysis as previously described by Ismail Suhaimy et al. [16]. Briefly, 1 mL MeOH was mixed thoroughly with 10 mg CEDL and then filtered through the 0.45 μm pore size filter membrane. The filtrate was then analyzed using a Waters Delta 600 with 600 Controller and Waters 2996 Photodiode Array (Milford, MA, USA) equipped with an autosampler, column heater and online degasser. A minibore Phenomenex Luna 5 mm C18 column (dimensions 250 × 4.60 mm) was used to separate CEDL at 27 °C using a one-step linear gradient. The elution system comprising of 0.1% aqueous formic acid (eluant A) and acetonitrile (eluant B) was set in the following conditions: Initial conditions of 5% B with a linear gradient reaching 25% B at 12 min and maintained for 8 min. The gradient was then lessened to 15% B at 22 min and sustained for another 8 min (30 min). The program was reverted to the early solvent arrangement at 35 min. Throughout the analysis, the flow rate and the injection volume of 1.0 mL/min and 10 μL was applied, respectively. The HPLC was observed at various wavelengths (i.e. 210, 254, 280, 300, 330 and 366 nm). The data obtained was assessed using Millenium 32 Software (Waters Product) that was installed into the system. Additional analysis was also done to compare the HPLC chromatogram of CEDL against several standard flavonoid-based compounds (e.g. quercetin, quercitrin, fisetin, naringenin, pinostrobin, genistein, hesperetin and flavanone).

#### UPLC-HRMS analysis of CEDL

The liquid chromatography analysis of CEDL was performed using an UPLC-HRMS system (Dionex Ultimate 3000, Thermo Scientific, USA) equipped with a quaternary gradient pump, an autosampler set at 4 °C, a thermostatically column compartment and a photodiode array detector. The UPLC system coupled to Thermo Q-Exactive orbitrap high resolution mass spectrometer (Thermo Scientific, USA) with ESI in negative ion mode and using full mass scan type. The separation was performed using C18 Cortecs column (100 mm × 2.1 mm i.d. 1.6 μm Waters) at 40 °C column temperature and the injection volume was 5 μL. Aqueous formic acid (0.1%, v/v, A) and acidic acetonitrile (0.1%, v/v, B) were used as the eluant system. The gradient elution was programmed at a flow rate of 0.3 mL/min as follows: initial conditions of 5% (v/v) B that increased linearly to 50% (v/v) B in 20 min. In addition, the spray voltage was set at 3.0 kV, the capillary temperature was 350 °C and S-lens RF level was fixed at 55 V to get the best experimental conditions. N_2_ was used as the Sheath gas with flow rate at 15 (arbitrary units), Aux gas with flow rate at 20 (arbitrary units) and sweep gas with flow rate at 5 (arbitrary units). The MS resolution was set at 17,000 and FTMS AGC target was 2e5, FT-MS/MS AGC target at 1e5, isolation width of 1.5 amu and the normalization collision energy at 35%. In addition, the maximum ion injection time was at 500 ms. the mass range used was in the range of 100–1000 amu (amu). The UPLC-HRMS system was controlled with Xcalibur 3.0 data system software.

### Antioxidant studies of CEDL

#### Total phenolic content

Total phenolic content (TPC) of CEDL was assessed using the Folin-Ciocalteu procedure based on the slightly modified method of Singleton and Rossi [17]. A mixture of CEDL (1 mg) and 1 ml of 80% methanol containing 1% hydrochloric acid and 1% distilled water (dH_2_O) was prepared and allowed to shake at 200 rpm for 2 h on a shaker at room temperature before being centrifuged at 6000 rpm for 15 min to acquire the supernatant. Approximately 400 μl Folin-Ciocalteu reagent was then added to 200 μl of the obtained supernatant and kept for 5 min at room temperature before the addition of 400 μl of 60 mg/ml sodium bicarbonate solution. The mixture was then left for 90 min at room temperature prior to the absorbance measurement at 725 nm. Later, the optical density (OD) of gallic acid (GA) standard was used to create a calibration curve and the level in the samples was expressed as GA equivalents (GAE)-TPC mg/100 g dry weight.

#### Diphenylpicrylhydrazyl radical scavenging assay

CEDL was also subjected to the diphenylpicrylhydrazyl (DPPH) radical scavenging assay based on the slightly modified method of Blois [18]. Approximately 1.0 mg/mL of the extract’s stock solution was successively diluted to obtain the final acquired concentrations of 200, 100, 50, 25, 12.5, 6.25, and 3.13 μg/mL. Then, 50 μL of each concentration of the prepared sample was added in 96-well microtiter plate in triplicates and mixed with 50 μL of 1 mM DPPH in ethanolic solution together with 150 μL of absolute ethanol. The plate was shaken (500 rpm; 15 s) and then left for 30 min at room temperature. The standard antioxidant used (positive control) in the present study was L-ascorbic acid (concentration range of 3.13–200 μg/mL). On the other hand, the negative control was prepared by adding 50 μL deionized water to 950 μL 100 μM DPPH reagent. The absorbance of all test solutions was then spectrophotometrically estimated at 520 nm and the results obtained were expressed as percentage inhibition (I%) using the following equation:

$$ I\%=\frac{\left({\mathrm{Abs}}_{\mathrm{control}}-{\mathrm{Abs}}_{\mathrm{sample}}\right)}{{\mathrm{Abs}}_{\mathrm{control}}}\mathrm{X}\kern0.5em 100\kern0.5em \mathrm{whereby}; $$

Abs_control_ refers to the absorbance of the control reaction with 50 μL deionized water alone, and Abs_sample_ refers to the absorbance after treatment with extract or ascorbic acid. The linear regression analysis of dose-response curve plotting between *I*% and concentrations was performed to determine the effective concentration of the sample required to scavenge DPPH radical by 50% (EC_50_).

#### Superoxide anion radical scavenging assay

CEDL was subjected to the superoxide anion (SOA) radical scavenging assay based on the slightly modified method of Chang et al. [19]. Briefly, 100 ml of 4.1 mM/L nitroblue tetrazolium (NBT) solution was prepared by adding 34.0 mg 4-NBT chloride, 3.15 g Tris-HCl, 15.0 mg 5-bromo-4-chloro-3-indolyl phosphate and 0.1 g MgCl2 to 100 ml dH_2_O. Then, 100 ml of a reaction mixture was made by adding 0.025 mM NBT solution with approximately 50.0 mg xanthine, 0.53 g NA2CO3 (pH 10.2) and 4.0 mg EDTA and then kept at 4 °C until used. The negative control group comprising of 999 ml of a reaction mixture was then placed into a microcuvette and kept in a 25 °C cell holder in a spectrophotometer to calibrate it to auto zero. Then, approximately 1.0 ml of XO (20 U/ml) was added to the reaction mixture and manually shake thoroughly for 5 s to generate the formation of superoxides. On the other hand, for the treatment groups, the extract or ascorbic acid solutions were mixed with 799 ml reaction mixture and assessed as mentioned above. Each mixture was then subjected to OD measurement using a Lambda 2S spectrophotometer at 560 nm for 120 s.

#### Oxygen radical absorbance capacity test

Oxygen radical absorbance capacity (ORAC) assay was used to assess the antioxidant capacity of CEDL according to the slightly modified procedure of Huang et al. [20]. Briefly, phosphate buffer solution (PBS; pH 7.4) was mixed with 175 μl of 160 μg/ml samples or blank (solvent/PBS) to dissolve it and then 25 μl of the mixture of samples or blank were added to the 96-well microplates followed by 150 μl of 1 mM fluorescent sodium salt solution. The plate was then incubated at 37 °C for 10 min after which 25 μl of 240 mM 2, 20-azobis (2-amidinopropane) dihydrochloride (AAPH) solution was supplemented to make up a total volume of 200 μl/well. Once AAPH has been added, the plate was shaken at the maximum intensity for 50 s to mix well the solutions. The fluorescence spectrophotometer (BMG FLUOstar Omega, UK) equipped with an automatic thermostatic autocell-holder was used to kinetically record the fluorescence colour formation every 1 min for 2 h at 37 °C until it reached 0 (excitation at 485 nm, emission at 528 nm). The data obtained were analysed using MARS Data Analysis Reduction 2.0 Software (BMG LABTECH, Germany). Trolox, in different concentrations, was applied as the reference standard and the ORAC value was articulated as mmol of Trolox Equivalents (TEs) per gram of dry weight.

### Experimental animals

Adult male *Sprague-Dawley* rats (180–220 g; 8–10 weeks) were purchased from a private company (Chenur Supplier, Seri Kembangan, Selangor, Malaysia). The animals were allowed to adapt under the standard husbandry conditions (27 ± 2 °C; 55 ± 10% relative humidity; 12 h light/dark cycle) in the Animal Holding Unit, FMHS, UPM and provided access to a sufficient amount of food and water supply ad libitum throughout the experimentation period. The experimentation protocol was consented and the ethical clearance was granted (Ethical approval no: UPM/FPSK/PADS/BR-UUH/00506) by the Animal Ethics Committee of Faculty of Medicine and Health Sciences (FMHS, UPM). The animals were handle based on the guidelines for the care of laboratory animals and the ethical guidelines for investigation of experimental pain in conscious animals adopted by the FMHS, UPM. Animals were fasted for 48 h before subject all assays.

### Hepatoprotective assay

#### PCM-induced hepatotoxicity test

Rats were fasted for 48 h before the start of the experiment and divided into six groups (*n* = 6), which were orally treated once daily with 8% Tween 80 (v/v) in dH_2_O (vehicle 1; normal control; Group 1), 8% Tween 80 (v/v) in dH_2_O (vehicle 1; negative control; Group 2), 50 mg/kg NAC (positive control; Group 3) or, 50, 250, or 500 mg/kg CEDL (treatment groups; Group 4–6) for 7 successive days. Table 1 summarized the experimental design of the present study. The selection of dose range for CEDL was made based on a previous suggestion by Schmeda-Hirschmann and Yesilada [21]. Three hours after the respective test solutions administration on Day 7th, 3 g/kg PCM was orally administered to all groups except for Group 1, which was administered with 8% Tween 80 (vehicle). Forty-eight hours after PCM administration, blood collection was made on diethyl ether-anesthetized rats for serum biochemical parameters investigation. After blood collection the rats were sacrificed by cervical dislocation and the liver was immediately collected for histopathological examination.

**Table 1:** Experimental design of the study on hepatoprotective effect of CEDL in PCM-induced rat models

Inducer	Group (*n* = 6)	Type of group	Administered with:
10% DMSO	1	Normal	10% DMSO
PCM	2	Negative	10% DMSO
	3	Positive	50 mg/kg NAC
	4	Treatment	50 mg/kg of CEDL
	5	Treatment	250 mg/kg of CEDL
	6	Treatment	500 mg/kg of CEDL

Normal – Group pretreated and treated with vehicle only; Negative – Group pretreated with vehicle and treated with PCM; Positive – Group pretreated with NAC and treated with PCM; Treatment – Group pretreated with CEDL and treated with PCM

#### Biochemical parameter assay

The collected blood (3 ml) was allowed to coagulate at 37 °C for 45 min followed by the process of centrifugation (3000 rpm; 10 min) to acquire the serum, which was later subjected to the determination of alanine aminotransferase (ALT), alkaline phosphate (ALP), aspartate aminotransferase (AST), total and direct bilirubin and total protein levels using the Hitachi 902 Automatic Chemical Analyser [14].

#### Histopathological study

The collected liver was washed with normal saline and fixed in 10% formalin solution. The tissues were then processed in an automated tissue processor for 14 h, which comprise of tissue dehydration in gradual ethanol (50–100%), tissue clearance in xylene, and, finally, tissue embedment in paraffin wax. The tissue was then cut into various sections of 5–6 μm thickness using a rotary microtome (Leica RM 2125 RTS, Singapore) followed by the staining process (hematoxylin and eosin (H&E staining) for microscopic observation [14]. Sections were then examined for histopathological changes following each treatment regimen and scored based on the severity of the liver injury according to the modified method of El-Beshbishy et al. [22]. Briefly, stained rats’ liver was scored for the respective presence of necrosis, inflammation and hemorrhage through examining under the light microscope (40 x magnification), using the following severity scoring criteria: 0 – normal; 1 – mild necrosis, inflammation or, hemorrhage; 2 – moderate necrosis, inflammation or, hemorrhage; and, 3 – severe necrosis, inflammation or, hemorrhage.

#### Assessment of the liver’s endogenous antioxidant enzymes activity

##### Preparation of liver homogenates

About 1 ml of cold PBS buffer was added to 100 mg of minced liver tissue and homogenized using a steel homogenizer to obtain the liver homogenate. The liver homogenate was centrifuged (4000 rpm; 25 min) at 4 °C using Sorvall™ Legend™ Micro 17R microcentrifuge (Thermo Fisher Scientific) to obtain the supernatant for antioxidant enzymes’ activity determination [14].

##### Superoxide dismutase activity

The superoxide dismutase (SOD) activity of the supernatants of liver tissue homogenates were determined according to the manufacturer’s procedure provided in the Cayman’s Assay Kit (Cayman Chemical, USA). Firstly, various concentrations of the SOD standard were prepared (0–0.05 U/ml). Then, 10 μl of each standard or CEDL, 200 μl of the diluted radical detector and 20 μl of diluted xanthine oxidase was sequentially added into each plate’s well to initiate the reactions. The plate was covered, incubated on a shaker at room temperature for 30 min followed by the absorbance measurement (440–460 nm) using an ELISA reader (Asys UVM 340, UK). The SOD activity, defined as the amount of enzyme needed to exhibit 50% dismutation of the superoxide radical, was expressed as units per gram tissue.

##### Catalase activity

The obtained supernatants of liver tissue homogenates were also subjected to the catalase (CAT) activity determination according to the manufacturer’s procedure provided in the Cayman’s Assay Kit (Cayman Chemical, USA). First, various concentrations of formaldehyde standard were prepared (0–75 μM). Then, 20 μl of each formaldehyde standard or CEDL was sequentially added into each well followed by methanol (30 μl), diluted assay buffer (100 μl) and, finally, diluted hydrogen peroxide (20 μl) to initiate the reactions. The plate was then covered, incubated on a shaker for 20 min at the room temperature being added with diluted potassium hydroxide (30 μl) to terminate the reaction. Later, CAT purpald (chromogen) (30 μl) was added into each well and incubated again on a shaker for 10 min at room temperature before catalase potassium periodate (10 μl) was finally added to each well and the plate was incubated on a shaker for 5 min at room temperature. Absorbance reading for each plate was taken at 540 nm using ELISA reader (Asys UVM 340, UK). The CAT activity, expressed as the amount of enzyme that caused the formation of 1.0 nmol of formaldehyde per min at 25 °C, was expressed in terms of unit per gram tissue.

### Statistical analyses

The data obtained were analyzed using the one-way ANOVA and the differences between treatment and control groups were determined using Dunnett’s post-hoc test. *p* < 0.05 is used as the limit of significance.

## Results

### Phytoconstituents content of CEDL

#### Qualitative phytoconstituents of CEDL following the phytochemicals screening

Following the qualitative phytochemical screening, CEDL revealed the presence of triterpenes and steroids as the major constituents followed by flavonoids (Table 2).

**Table 2:** The phytochemical constituents of CEDL

Type of extract	Types of phytochemical constituents	Scoring of bioactive compounds
	Alkaloids	–
	Saponins	–
	Flavonoids	1+
CEDL	Tannins	–
	Triterpenes	3+
	Steroids	3+

**Note that:** - – Not detected, 1+ − Weak colour, 2+ − Mild colour, 3+ − Strong colour

#### Phytochemicals profile of CEDL following the HPLC analysis

**Fig. 1:**
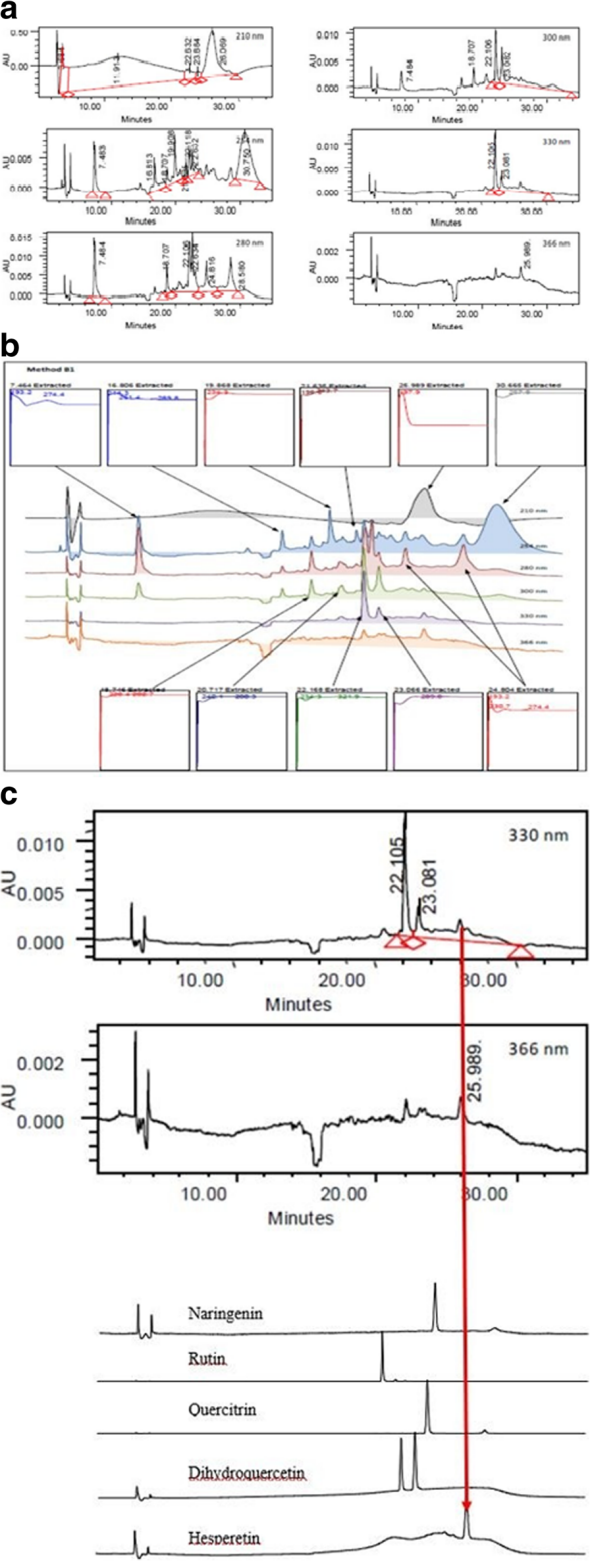
**a** HPLC profile of CEDL at various wavelengths. **b.** UV spectral analysis of various peaks detected following the HPLC analysis of CEDL. **c.** Comparison of chromatograms between CEDL and several pure flavonoids revealed the presence of only hesperetin

HPLC profile of CEDL at various wavelengths is shown in Fig. 1a. Two peaks were detected at the wavelength of 330 and 366 nm with the recorded retention time (RT) of 22.105 (Peak 1) and 23.081 (Peak 2) min. Further analysis of the UV spectra of these peaks revealed that only Peak 1 possessed a wavelength of 234.3 nm (Band A) and 321.9 nm (Band B) (Fig. 1b). Further investigation was performed to compare the chromatogram of CEDL against the respective chromatogram of several known bioactive compounds revealed the presence of only hesperetin in CEDL (Fig. 1c).

#### Phytochemical profile of CEDL following the UPLC-HRMS analysis

**Fig. 2:**
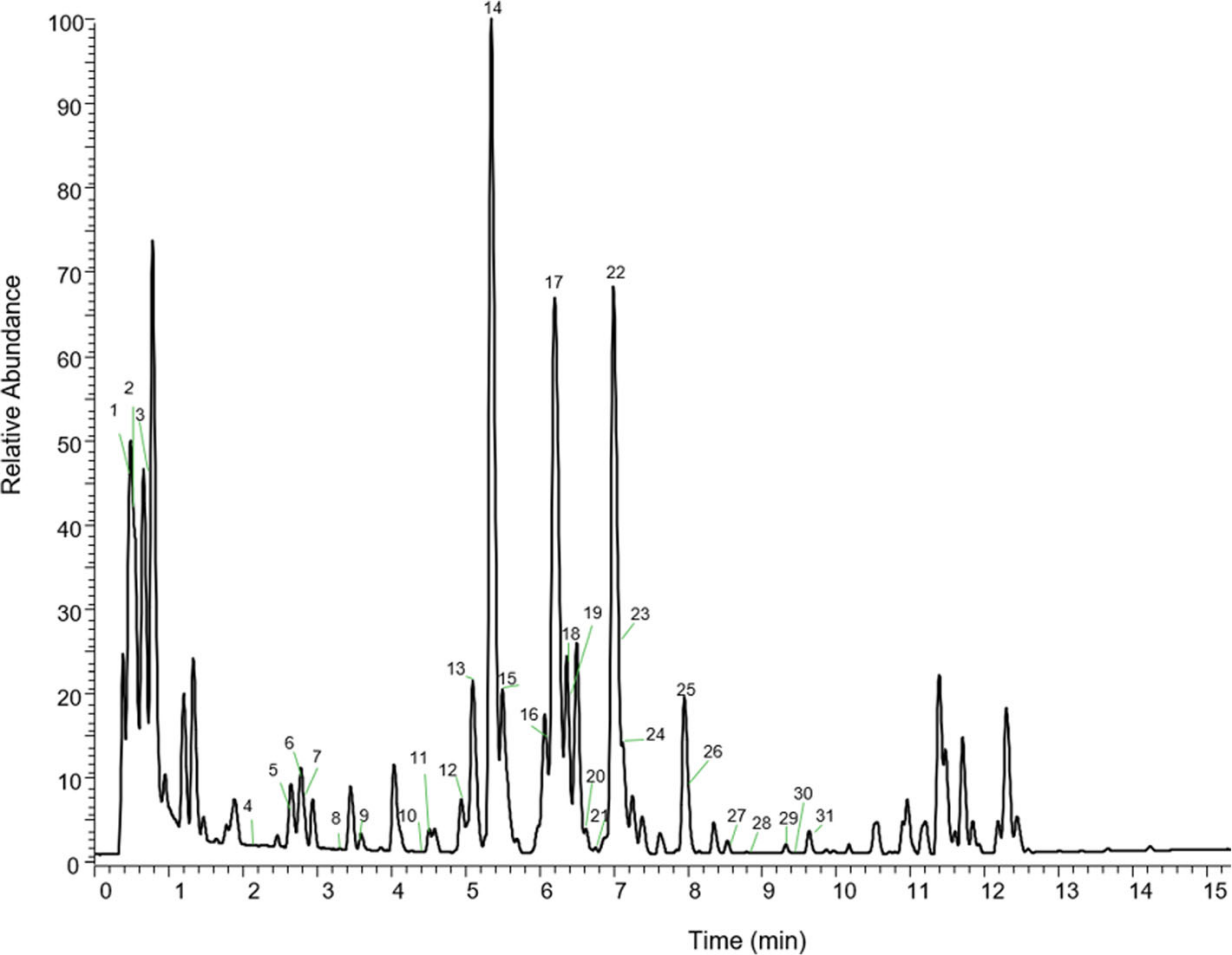
UPLC-HRMS profile of CEDL in negative ion mode

The phytoconstituents of CEDL after UPLC-HRMS analysis is shown in Table 3*.* A total of 30 compounds belonging to the class of polyphenols were identified, namely hydroxybenzoic acids, hydroxycinammates and flavonoid groups (Fig. 2). Some of these compounds, namely apigenin, rutin, kaempferol, quercetin and hesperidin, play a vital part in the antioxidative defence system against oxidative stress.

**Table 3:** Peak assignments of metabolites using UPLC-HRMS of D.linearis chloroform extract negative ion mode

Peak no	Tentative identification	t_**R**_ (min)	Molecular formula	Exact mass [M-H]^**−**^	∆ Mass (ppm)
1	Quinic acid	0.60	C_7_H_11_O6	191.05542	2.122
2	Citric acid	0.62	C_6_H_7_O_7_	191.01913	2.623
3	protocatechuic acid-4-O-*β*-hexoside	0.77	C_13_H_15_O_9_	315.07294	5.972
4	Galloylquinic acid	2.07	C_14_H_15_O_10_	343.06763	4.830
5	Hesperidin	2.62	C_28_H_33_O_15_	609.18384	4.011
6	Coumaryl-hexoside	2.82	C_15_H_17_O_8_	325.09341	4.971
7	Caffeic acid	2.83	C_9_H_7_O_4_	179.03432	2.429
8	Ferulic acid	3.27	C_10_H_9_O_4_	193.05011	2.977
9	Catechin	3.61	C_15_H_13_O_6_	289.07169	3.547
10	p-coumaric acid	4.44	C_9_H_7_O_3_	163.03922	3.063
11	Quercetin-3,7-diglucoside	4.53	C_27_H_29_O_17_	625.14197	3.270
12	Dichotamain B	4.98	C_21_H_23_O_12_	467.12036	4.191
13	Rutin isomer i	5.11	C_27_H_29_O_16_	609.14655	2.526
14	Rutin isomer ii	5.38	C_27_H_29_O_16_	609.14612	1.82
15	Isoquercetrin	5.52	C_21_H_19_O_12_	463.08871	3.882
16	Kaempferol-3-*O*-galactoside	6.16	C_21_H_19_O_11_	447.09384	3.695
17	Diosmetin 6,8-di-*C*-glucoside	6.22	C_28_H_31_O_16_	623.12101	0.56
18	Quercetin isomer i	6.23	C_15_H_9_O_7_	301.03598	5.650
19	Chysoeriol-6,8-di-*C*-glucoside	6.24	C_28_H_31_O_16_	623.16107	0.656
20	Dichotomain A-isomer i	6.74	C_23_H_25_O_13_	509.13135	4.680
21	(+) aromadendrin	6.77	C_15_H_11_O6	287.05646	5.035
22	Kaempferol isomer i	7.01	C_15_H_9_O_6_	285.04037	3.528
23	Apigenin-8-*O*-glucoside	7.03	C_21_H_19_O_9_	431.09842	2.660
24	Diosmetin 6,8-di-C-glucoside	7.15	C_22_H_21_O_11_	461.10962	3.865
25	Quercetin isomer ii	7.94	C_15_H_9_O_7_	301.03555	4.222
26	Dichotomain A- isomer ii	7.97	C_23_H_25_O_13_	509.13116	4.307
27	Kaempferol-3-O-glucoside	8.45	C_30_H_25_O_13_	593.12970	1.236
28	Apigenin	8.89	C_15_H_9_O_5_	269.04578	4.944
29	Kaempferol isomer ii	9.27	C_15_H_9_O_7_	285.04074	4.826
30	Cleomiscosin isomer i	9.30	C_20_ H_17_O_8_	385.09348	3.963
30	Cleomiscosin isomer ii	9.61	C_20_ H_17_O_8_	385.09341	4.197

### Antioxidant property and activity of CEDL

#### TPC value of CEDL

The TPC value of CEDL was measured to be approximately 15 mg/100 g GAE indicating a very low content of total phenolic compounds (Table 4). For comparison purposes, it is worth-highlighting that compound(s) with the TPC value of more than 1000 mg GAE/100 g is considered to possess the highest TPC value.

**Table 4:** Antioxidant potential of CEDL

Treatment group	Antioxidant activity			
	TPC value (mg/100 g GAE)	DPPH (% inhibition)	SOA (% inhibition)	ORAC value (TE/100 g)
200 μg/ml CEDL	14.8 ± 2.1	15.2 ± 0.0	28.9 ± 2.5	46,960.0 ± 36.0

1) Data presented are mean value of triplicate wells in duplicate experiments; SEM < 5%

2) Data for TPC analysis is expressed as TPC mg/100 g GAE

TPC value > 1000 mg GAE/100 g is considered as having a high TPC content.

TPC value is expressed as mg equivalent to gallic acid per 100 g of dry weight (mg GAE/100 g).

3) Data for DPPH and SOA radical scavenging assays are expressed as percentage of radial scavenging activity (% inhibition).

High: 70–100%; Moderate: 50–69%; Low: 0–49%

4) Data for ORAC assay is expressed as ORAC value, which is μmol equivalent to Trolox per 100 g of dry weight (μmol TE/100 g).

#### Effect of CEDL on DPPH radical scavenging assay

CEDL (200 μg/ml) exerted a lack of antioxidant activity as indicated by the low percentage of radical scavenging activity (≈15%) when assessed using the DPPH radical scavenging assay (Table 4). Therefore, no IC_50_ value was recorded.

#### Effect of CEDL on SOA radical scavenging assay

CEDL (200 μg/ml) exhibited a lack of antioxidant activity as indicated by the low percentage of radical scavenging activity (< 30%) when assessed using the SOA radical scavenging assay (Table 4).

#### Effect of CEDL on ORAC assay

CEDL (200 μg/ml) exerted a remarkable antioxidant capacity with the recorded ORAC value of approximately 47,000 μmol TE/100 g dry weight when assessed using the ORAC antioxidant capacity assay (Table 4).

### Hepatoprotective effect of CEDL against PILI

#### Effect of CEDL on body and liver weights of PCM-intoxicated rats

Changes in the liver weight (LW), body weight (BW), and liver/body weight (LW/BW) ratio following the administration of CEDL into rats intoxicated with PCM are shown in Table 5. Rats treated with PCM alone (negative group) demonstrated significant (*p* < 0.05) increased in the LW, but not BW, in comparison to the untreated rats (normal group). Pretreatment of rats with 50 mg/kg NAC or 500 mg/kg, but not 50 and 100 mg/kg, CEDL caused significant (*p* < 0.05) attenuation of the PCM-induced toxic effects specified by the reduced in LW and LW/BW ratio.

**Table 5:** Effect of CEDL, in the dose range of 50 to 500 mg/kg, on the body and liver weights, and liver/body weight ratio of PCM-intoxicated rats

Treatment	Dose (mg/kg)	Body weight (BW) (g)	Liver weight (LW) (g)	LW/BW (%)
Normal	–	204.3 ± 3.2	6.1 ± 0.7	2.99
Negative	–	206.2 ± 4.9	9.1 ± 0.2^a^	4.41^a^
Positive	50	204.9 ± 6.6	6.4 ± 0.5^b^	3.12^b^
CEDL	50	201.7 ± 4.2	8.0 ± 1.1	3.97
	250	204.4 ± 5.1	7.1 ± 0.1^b^	3.47^b^
	500	202.8 ± 6.3	6.0 ± 0.6^b^	2.96^b^

Normal – Group pretreated and treated with vehicle only; Negative – Group pretreated with vehicle and treated with PCM; Positive – Group pretreated with NAC and treated with PCM; CEDL – Group pretreated with CEDL and treated with PCM

^a^ Data differed significantly (p < 0.05) when compared against the normal group

^b^ Data differed significantly (*p* < 0.05) when compared against the negative control group

#### Effect of CEDL pretreatment on the serum liver AST and ALT level of PCM intoxicated rats

**Fig. 3:**
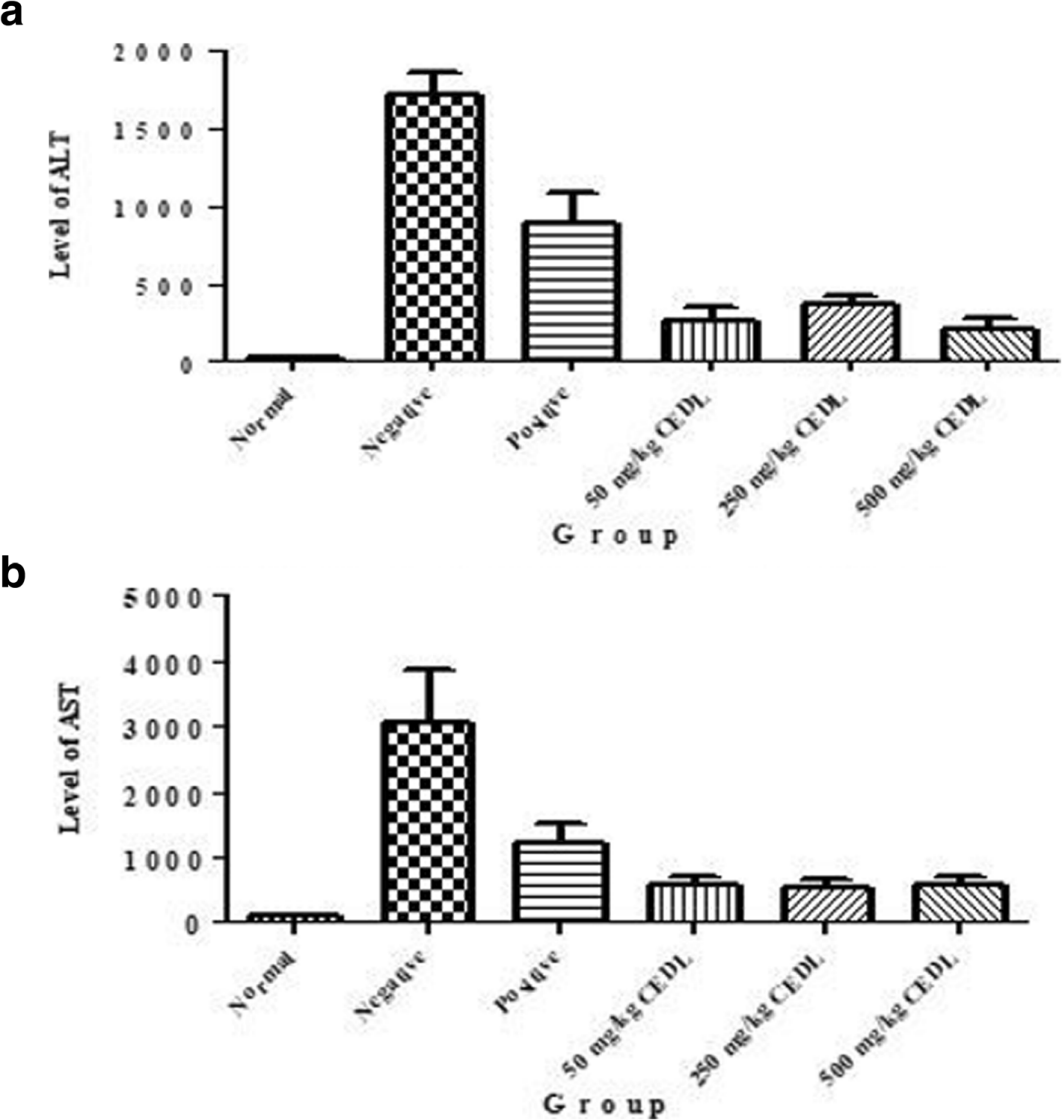
**a** Effect of CEDL on the serum level of AST in the PCM-induced liver damage rats. **b.** Effect of CEDL on the serum level of ALT in the PCM-induced liver damage rats

Serum level of two of the liver’s enzymes, namely ALT and AST, following pretreatment of PCM-intoxicated rats with CEDL, is illustrated in Fig. 3 a and b. The level of AST and ALT was significantly (*P* < 0.05) higher in PCM-intoxicated rats (negative group) in comparison to the untreated group (normal group) indicating the occurrence of liver damage. On the other hand, pretreatment of PCM-intoxicated rats with CEDL or NAC significantly (*P* < 0.05) reduced the serum level of both enzymes in comparison to the negative group.

#### Histopathological findings on the architecture of liver tissues of PCM-intoxicated rats after pretreatmnet with CEDL

**Fig. 4:**
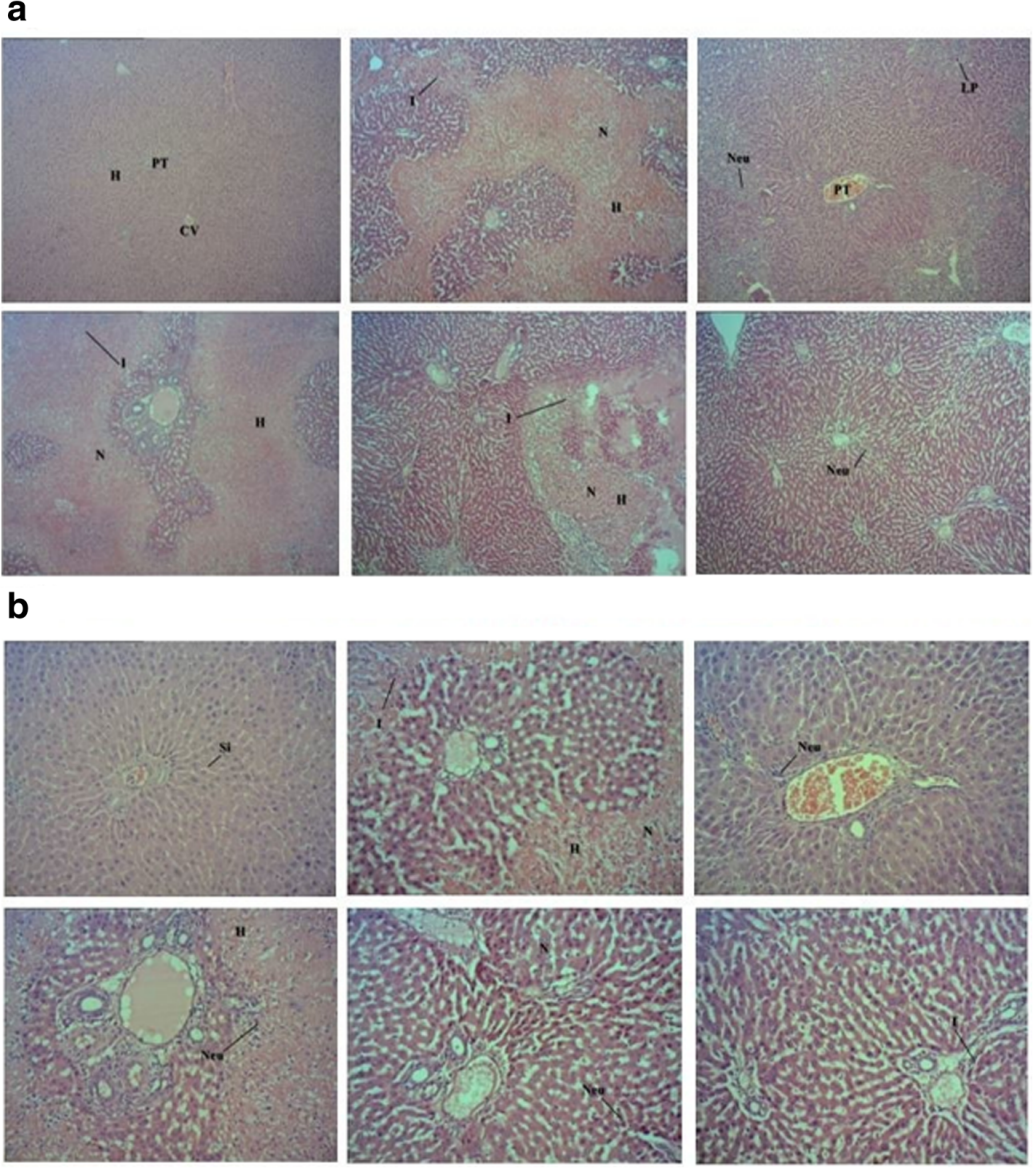
Histopathological observations of PCM-intoxicated liver tissues of rats pretreated with CEDL or NAC at magnification 40x **a** and 100x **b**. The liver tissue of normal group shows normal liver architecture, which was damaged by 3 g/kg PCM intoxication as indicated by the occurrence of massive necrosis **(N)**, haemorrhage **(H)**, and inflammation **(I)**. Pretreatment with 5- mg/kg NAC reversed the PCM induced toxicity in the liver tissue by causing mild **I** evidence by the presence of neutrophils **(Neu)** and lymphoplasmacytic **(LP)** cells while the portal tract exerted mild **I** indicated by the presence of scattered **Neu**. On the other hand, pretreatment with 50 mg/kg CEDL failed to reverse the effect of PCM as indicated by the presence of a severe **H** with area of **N** with the infiltration of **I** cells and the present of **Neu** cells. Pretreatment with 250 mg/kg CEDL caused mild **I** in the liver tissue as indicated by the presence of lymphocytic cells **(L)** with no area of **N** and **H** with the portal tract showing the presence of scattered **I** cells at the liver parenchyma. Lastly, pretreatment with 500 mg/kg CEDL successfully reversed the toxic effect of PCM on liver tissue as indicated by the absence of **H**, **N** and **I** while the portal tract only showed a mild infiltration of **Neu** at the liver parenchyma

Figure 4a and b show the histopathological findings of liver tissues of PCM-intoxicated rats following pretreatment with CEDL or NAC. Tissue sections of the normal group showed the normal liver architecture indicate by the presence of normal hepatic cells with the characteristic morphology of well-preserved cytoplasm, prominent nucleus, sinusoidal spaces and mostly visible vein. However, tissue sections of the negative control group demonstrated severe architectural damage specified by the presence of marked centrilobular necrosis, hemorrhage, inflammation and inflammatory exudates, and steatosis, which is due to the toxic effect of PCM. Pretreatment with 250 and 500 mg/kg CEDL and 50 mg/kg NAC attenuated the toxic effect of PCM and reduced the architectural damage to the liver. Tables 6, 7, 8 (A-C) shows the effect of CEDL on histopathological scoring of the liver tissue of rats intoxicated with PCM.

**Table 6:** Tissue necrosis scoring of PCM-intoxicated rat’s liver following the oral treatment with vehicle (10% DMSO), 50 mg/kg NAC or, 50, 250 and 500 mg/kg CEDL in comparison to the normal tissue

Group	Dose	Number of animals	Severity score of tissue necrosis				Mean ± SEM
	mg/kg	n	0	1	2	3	
Normal	–	6	6	0	0	0	0
Negative	–	6	0	0	0	6	3.00 ± 0.00
Positive	50	6	0	5	1	0	1.17 ± 0.17***
CEDL	50	6	0	0	1	5	2.83 ± 0.17
	250	6	0	4	2	0	1.33 ± 0.21***
	500	6	5	1	0	0	0.17 ± 0.17***

Note: The severity score of tissue necrosis was expressed as Mean ± SEM for 6 animals. One-way ANOVA was followed by Dunnett’s *post-hoc* test, ***p < 0.001 vs. vehicle

**Table 7:** Tissue inflammation scoring of PCM-intoxicated rat’s liver following the oral treatment with vehicle (10% DMSO), 50 mg/kg NAC or, 50, 250 and 500 mg/kg CEDL in comparison to the normal tissue

Group	Dose	Number of animals	Severity score of tissue inflammation				Mean ± SEM
	mg/kg	n	0	1	2	3	
Normal	–	6	6	0	0	0	0
Negative	–	6	0	0	1	5	2.83 ± 0.17
Positive	50	6	3	3	0	0	0.50 ± 0.22***
CEDL	50	6	0	0	0	6	3.00 ± 0.00
	250	6	4	2	0	0	0.33 ± 0.21***
	500	6	5	1	0	0	0.17 ± 0.17***

Note: The severity score of tissue inflammation was expressed as Mean ± SEM for 6 animals. One-way ANOVA was followed by Dunnett’s *post-hoc* test, ***p < 0.001 vs. vehicle

**Table 8:** Tissue hemorrhage scoring of PCM-intoxicated rat’s liver following the oral treatment with vehicle (10% DMSO), 50 mg/kg NAC or, 50, 250 and 500 mg/kg CEDL in comparison to the normal tissue

Group	Dose	Number of animals	Severity score of tissue hemorrhage				Mean ± SEM
	mg/kg	n	0	1	2	3	
Normal	–	6	6	0	0	0	0
Negative	–	6	0	0	0	6	3.00 ± 0.00
Positive	50	6	1	3	2	0	1.17 ± 0.31***
CEDL	50	6	0	0	2	4	2.67 ± 0.21
	250	6	0	2	3	1	1.83 ± 0.31**
	500	6	6	0	0	0	0.17 ± 0.17***

Note: The severity score of tissue hemorrhage was expressed as Mean ± SEM for 6 animals. One-way ANOVA was followed by Dunnett’s *post-hoc* test, ***p < 0.001, **p < 0.01 vs. vehicle

#### Effect of CEDL on the liver endogenous enzymatic antioxidant system activity of PCM-intoxicated rats

Table 9 shows the effect of CEDL on the liver endogenous antioxidant enzymes activity of PCM-intoxicated rats. There was a significant (*p* < 0.05) reduction in the activity of CAT and SOD following the oral administration of PCM (negative control group) in comparison to the normal group. However, the decrease level of CAT and SOD resulting from the noxious effect of PCM was significantly (p < 0.05) reversed following pretreatment with 50 mg/kg NAC or, 250 and 500 mg/kg CEDL.

**Table 9:** Effect of CEDL pretreatment on the endogenous antioxidant enzyme system activity, namely CAT and SOD, in the liver of PCM-induced liver intoxication in rats

Treatment	Dose (mg/kg)	CAT (U/g tissue)	SOD (U/g tissue)
Normal	–	201.6 ± 10.7	167.2 ± 8.1
Negative	–	92.4 ± 8.1^a^	82.6 ± 9.7^a^
Positive	50	151.6 ± 9.7^ab^	129.3 ± 6.6^ab^
CEDL	50	96.3 ± 8.8^a^	91.9 ± 9.2^a^
	250	129.6 ± 5.2 ^ab^	122.1 ± 4.9^ab^
	500	193.3 ± 5.8^ab^	152.6 ± 6.9^ab^

Normal – Group pretreated and treated with vehicle only; Negative – Group pretreated with vehicle and treated with PCM; Positive – Group pretreated with NAC and treated with PCM; CEDL – Group pretreated with CEDL and treated with PCM

Values are expressed as means ± SEM of six replicates

^a^ Data differed significantly (*p* < 0.05) when compared against the normal group

^b^ Data differed significantly (*p* < 0.05) when compared against the negative control group

## Discussion

CEDL ability to exert hepatoprotective activity against PILI in rats were investigated in this study. Liver function test, liver antioxidant enzyme levels and histological studies were performed to assess hepatoprotective properties of this plant. The results obtained show that overdose PCM administration altered various liver parameters by increasing the: i) LW and LW/BW ratio, and; ii) serum level of ALT and AST while decreasing the activity of several endogenous antioxidant enzymes, namely CAT and SOD. These findings were supported by histopathological observations, which show that PCM-induced severe degrees of histological damage to the liver tissue architecture due to the presence of necrosis, hemorrhage and inflammation.

It is widely known that PCM, at high doses, produces acute toxic effects that can result in PILI. This condition is attributed to PCM bioactivation to a toxic electrophile, *N*-acetyl *p*-benzoquinoneimine (NAPQI), which binds covalently to tissue macromolecules possibly triggering the oxidation of lipids or the perilous sulfhydryl groups (protein thiols) leading to the alteration in calcium homeostasis [23]. Immense generation of reactive species might cause a diminution of protective physiological moieties, thus, initiating injury to the macromolecules in vital biomembranes that leads to liver injury [24].

Liver enzymes (e.g. ALT and AST) and several other molecules, which are originally present in the cytoplasm of liver cells, leak into the bloodstream during liver injury resulting in the elevation of their level during blood test; thus, can and assist as an indicator for the liver injury [25]. Unusually high levels of serum ALT and AST observed in the PCM-intoxicated group seen in the present study indicate PCM-induced liver dysfunction and denote injury to the hepatocytes. Interestingly, pretreatment with CEDL reversed the increased serum enzymes in the PILI group suggesting the extract potential to prevent the intracellular enzymes leakage through its membrane-stabilizing activity. Histopathological findings corroborate well with the biochemical results suggesting that CEDL significantly contribute to the reduction of degree of injury induced by PCM.

Antioxidant enzymes reduced the toxic effect caused by superoxide anion by scavenging those free radicals to form hydrogen peroxide. Two of the vital enzymes in the enzymatic antioxidant defense system are CAT and SOD [26] and reduction in their activities have been reported to lead to several toxic effects. In the present study, it was observed that PILI caused a marked decrease in the activity of both enzymes whereas pretreatment with CEDL increased the hepatic SOD and CAT activities in PILI rats. This finding suggested that CEDL contained bioactive compounds that can modulate the endogenous enzymatic antioxidant enzymes and help prevents liver injury.

Other than that, CEDL has also been found in the present study to exert a high antioxidant capacity as proven using the ORAC assay despite its low TPC value and low free radical scavenging effects as shown using the DPPH and SOA assays. The contradictory findings between the ORAC assay against the DPPH- and SOA-assay could be partly attributed to the various mode of antioxidant actions reported elsewhere. Other than acting as a radical scavenger, other bioactive compounds have also been reported to exert antioxidant action by acting as electron donor, hydrogen donor, singlet oxygen quencher, peroxide decomposer, an enzyme inhibitor, metal-chelating agents, and synergist [27]. Concerning the mechanisms of action involved, Lobo et al. [27] have also cited two principal mechanisms via which antioxidants work, namely: i) a chain-breaking mechanism that involves donation of an electron to the free radical existing in the systems by primary antioxidant, and; ii) a mechanism that involves a quenching chain-initiating catalyst removing the ROS/reactive nitrogen species initiators (secondary antioxidants). Besides, some other bioactive compounds also can stimulate the synthesis of endogenous antioxidant molecules in the cell [28]. The presence of various modes of antioxidant actions might, therefore, explained the extract’s high antioxidant capacity that helps to reduce oxidative damage to the tissues as well as improving the activity of hepatic antioxidant enzymes. On the other part, the low free radical scavenging activity of CEDL could be attributed to the presence of triterpenes as its major constituents. Interestingly, Arora et al. [29] have earlier reported that *Centella asiatica*’s extract enriched with triterpenes resulted in a loss of free radical scavenging potential, thus, could be used to justify the low free radical scavenging potential of CEDL. Concomitantly, Castellano et al. [30] also reported that the data available currently concerning the antioxidant activity of triterpenes are rather inconsistent with various reports on the ability of triterpenes to react with diverse radical species resulted in a collection of varied results [31–34].

Although phenolic compounds are present in CEDL, the number of phenolic compounds detected was low based on the TPC value recorded. The low TPC value in CEDL might be attributed to the occurrence of only hydrophobic flavonoids in the extract with the absence of tannins. Moreover, reports have shown the link between the level of TPC and the intensity of free radical scavenging activity [35]. Phenolic compounds have been widely known to possess redox properties due to the existence of hydroxyl groups. Being good electron donors, these hydroxyl groups are responsible for facilitating free radical scavenging action [36]. Concurrent with the link described above, the low free radical scavenging activity of CEDL could, therefore, be attributed to the low TPC value.

The presence of triterpenes followed by flavonoids as major constituents of CEDL might strongly contribute to the strong antioxidant capacity but low free radical scavenging activity, activation of the endogenous enzymatic antioxidant system and protection against PILI. The ability of triterpenes: i) to exert hepatoprotective against PILI [37, 38], ii) to show antioxidant capacity via ORAC with low free radical scavenging activity [30, 39], and; iii) to activate the endogenous enzymatic antioxidant system [40] as seen with CEDL have been previously established. With regard to the antioxidant potential of CEDL, the antioxidant power of triterpenes is subject to debate since it is evidently affected by the singularities of the experimental systems engaged in its evaluation. For examples, oleanolic acid was found to capture ABTS+ radicals in a moderate-, dose-dependent-manner but cannot scavenge DPPH species. The latter observation is in agreement with the present study that shows CEDL lack of scavenging effect against the DPPH species. On the other hand, Castellano et al. [30] also reported on the ability of triterpenes like oleanolic acid to moderately captures the peroxyl radicals generated in the ORAC assay, which is also seen in the present study. Other than that, flavonoids have also been reported: i) to ameliorate PILI [41, 42], ii) to exert free radicals scavenging activity and marked antioxidant capacity [43, 44], and; iii) to improve the endogenous enzymatic antioxidant system, particularly CAT and SOD [45, 46].

Concerning the different in the antioxidant effect of flavonoids when measured against the DPPH radical scavenging assay or ORAC assay, Roy et al. [43] reported that certain flavonoids such as epicatechin showed a higher ORAC reading than epigallocatechin gallate but, on the contrary, epigallocatechin gallate demonstrated a stronger DPPH radical scavenging activity than epicatechin. These differences were further explained through the structure-activity relationship investigation, which shows that the lower ORAC value of epigallocatechin and epigallocatechin gallate is attributed to the OH replacement at the 3′ position in pyrogallol moieties when compared to their non-3′-OH counterparts (such as epicatechin and epicatechin gallate) [43]. However, the number of OH replacements is suggested to poorly correlate with the recorded ORAC value, but significantly affected the DPPH radical scavenging activity. Thus, the presence of flavonoids with non-3′-OH replacement in pyrogallol moieties in CEDL is postulated to contribute to the high ORAC value but low DPPH radical scavenging activity.

Preliminary qualitative phytochemical screening of CEDL demonstrated the presence of flavonoids. HPLC analysis of CEDL revealed at least two peaks showed the UV spectral that were characteristic of flavonoid-based bioactive compounds while comparison between the chromatogram of CEDL and several pure flavonoid standards revealed the presence of only hesperetin in the extract. Further analysis of CEDL using the UHPLC-HRMS procedure revealed the presence of 30 polyphenols, which were identified to belongs to the hydroxybenzoic acids, hydroxycinammates and flavonoid groups. Despite their presence in CEDL, these polyphenols contribute to low TPC content in CEDL and lack of free radical scavenging activity of CEDL and the latter effect of flavonoids has been discussed earlier. However, their presence and synergistic action also could be used to explain the significant antioxidant activity of CEDL as measured using the ORAC assay and hepatoprotective activity of CEDL as observed in the in vivo study.

Other than that, Vijayakumari and Leon Stephen Raj [47] have recently performed the GC-MS analysis on CEDL collected from Marthandam, Kanyakumari district, India in which the GC-MS spectrum of CEDL demonstrated the presence of 9 different major peaks representing 1H-inden-1-ol, 2,3-dihydro, phenol 2,5-bis(1,1-dimethylethyl)-, heptacosane, di-n-decylsulfone, ethanone, 2-(2- benzothiazolylthio)-1-(3,5-dimethylpyrazolyl)-, 1-bromoeicosane, methoxyacetic acid, 2-tridecyl ester, octadecane, 3-ethyl-5-(2-ethylbutyl)- and 1,2,4-benzene tricarboxylic acid, 4-dodecyl dimethyl ester. Interestingly, some of these compounds, namely phenol,2,5-bis(1,1-dimethylethyl)-, ethanone,2-(2-benzothiazolylthio) -1-(3,5-dimethyl pyrazolyl)-, and octadecane,3-ethyl-5-(2-ethyl butyl)-1,2,4-benzene tricarboxylic acid, 4-dodecyl dimethyl ester, have been reported to exert antioxidant activity [47]. It is believed that these compounds together with the triterpenes and flavonoids act synergistically to modulate the antioxidant properties of CEDL. The high antioxidant capacity of CEDL may help explain the significant hepatoprotective activity of the extract by counteracting the redox state precipitated intracellularly, hence, ensure hepatoprotection against PILI.

## Conclusions

Lipid-soluble phytoconstituents of *D. linearis* possess hepatoprotective activity, which is attributed partly to its high antioxidant capacity and also to the synergistic action of its phytoconstituents, namely triterpenes and flavonoids. This supports the potential use of *D. linearis* leaf as an antidote for the treatment of liver injury.

## Data Availability

The supporting materials can be obtained upon request via email to the corresponding author.

## Abbreviations

AAPH: 20-azobis (2-amidinopropane) dihydrochloride
Abs: Absorbance
ALP: Alkaline phosphate
ALT: Alanine aminotransferase
ANOVA: Analysis of variance
AST: Aspartate aminotransferase
BW: Body weight
CAT: Catalase
CEDL: Chloroform extract of *Dicranopteris linearis* leaves
CYP450: Cytochrome P450
DMSO: Dimethyl sulfoxide
DPPH: Diphenylpicrylhydrazyl
FMHS: Faculty of Medicine and Health Sciences
FRIM: Forest Research Institute Malaysia
GA: Gallic acid
GAE: Gallic acid equivalent
GSH: Reduced glutathione
H&E: Hematoxylin and eosin
HOX: Hypohalous acids
IBS: Institute of Bioscience
LW: Liver weight
NAC: *N*-acetylcysteine
NAPQI: *N*-acetyl-*p*-benzoquinone imine
NBT: Nitroblue tetrazolium
NO2: Nitrogen oxide
OD: Optical density
ORAC: Oxygen radical absorbance capacity
PBS: Phosphate buffer solution
PCM: Paracetamol
PILI: PCM-induced liver injury
SOA: Superoxide anion
SOD: Superoxide dismutase
TE: Trolox equivalent
TPC: Total phenolic content
UPM: Universiti Putra Malaysia
UV: Ultra violet
XO: Xanthine oxidase
